# Socio-cultural effects on children's initiation of joint attention

**DOI:** 10.3389/fnhum.2012.00286

**Published:** 2012-10-25

**Authors:** Yana Gavrilov, Sarit Rotem, Renana Ofek, Ronny Geva

**Affiliations:** The Developmental Neuropsychology Lab, Department of Psychology, The Gonda Multidisciplinary Brain Research Center, Bar Ilan UniversityRamat Gan, Israel

**Keywords:** joint attention, cross-cultural differences, tradition, gender, affect, play, exploration, social

## Abstract

Exchanging gazes with a social partner in response to an event in the environment is considered an effective means to direct attention, share affective experiences, and highlight a target in the environment. This behavior appears during infancy and plays an important role in children's learning and in shaping their socio-emotional development. It has been suggested that cultural values of the community affect socio-emotional development through attentional dynamics of social reference (Rogoff et al., 1993). Maturational processes of brain-circuits have been found to mediate socio-cultural learning and the behavioral manifestation of cultural norms starting at preschool age (Nelson and Guyer, 2011). The aim of the current study was to investigate the relations between cultural ecology levels and children's joint attention (JA). Initiation of JA bids was studied empirically as a function of the level of social load of the target toy (3 levels), the community level of adherence to traditional values (3 levels), parental education (2 levels), and gender. Sixty-two kindergarten aged children were enrolled in a structured toy-exploration task, during which they were presented with toys of various social loads, with social agents (i.e., mother and experimenter) present nearby, and non-social distracters presented intermittently. Measurements included the child's number of JA bids and the extent of positive affect. Analysis of variance indicated that the child's initiation of JA toward the social partner was affected by all levels of cultural ecology (i.e., toy's social load, adherence to tradition values, parental education, gender), thus supporting the study's hypotheses. The effects were such that overall, children, particularly girls' JA initiation was augmented in social toys and moderated by the socio-cultural variables. These results suggest that cultural ecology is related to children's JA, thereby scaffolding initiation of social sharing cues between children and adults. JA plays a role in adjusting children's internal representations of their respective ecological environment.

## Introduction

The need to share attention and experience among individuals that interact with each other is an intrinsic social need that accompanies humans throughout their lifespan, so as to organize their social and emotional experiences (Trevarthen and Aitken, 2001; Hobson, 2002). The behavior of social referencing in the presence of a third object or event, coined as joint attention (JA), is usually intertwined with a behavior of solitary exploration and is known to be a basic form of communication (Mundy and Sigman, 2006; Mundy and Jarrold, 2010). This ability involves the initiation of JA; namely, directing the partner's attention to some aspect of one's experience and a response to such an initiation, primarily by gaze following (Scaife and Bruner, 1975; Mundy et al., 2007). The process of JA involves a continued monitoring of one another's attention, and the awareness that this attention is being monitored by a social agent (Tomasello, 1995). In order for an interaction to be considered as JA, the social agents must show awareness to both the object and the partner, as well as to the shared perception that characterizes the situation so that activities surrounding the object are coordinated (Tomasello, 2005).

The ability to coordinate attention toward a third object in a dyadic interaction appears in the first year of the child's life and continues to develop as the child grows (Moll and Tomasello, 2004). JA behaviors start to appear as early as 3 months of age and elaborate for the next 15 months (Tomasello, 2005; Mundy and Jarrold, 2010). These behaviors include the ability to follow a social partner's direction of attention, i.e., responding to JA, and to direct the partner's attention to a certain aspect of the environment with the intention of sharing the experience, i.e., initiation of JA or JA bids (Mundy and Acra, 2006). Several frameworks of JA view this behavior as a primary communication mechanism which serves as a pivotal building block in the development of social competence (Bretherton, 1991; Baldwin, 1995; Mundy, 1995, 2003; Tomasello, 1995; Dawson et al., 2001; Mundy and Neal, 2000; Brauer et al., 2005). According to these frameworks, the ability to jointly attend to an object serves as a basis for the sharing of information and the understanding of mutual meanings and intentions (Mundy and Jarrold, 2010). We would therefore like to suggest that JA may reflect an evolving representation of the other agent's mind, abilities, and cultural values.

JA is known to be one of the first steps toward understanding the concept that people are independent agents with individual thoughts, beliefs, intentions, and points of reference. The goal of JA is to share intentions—concrete at the beginning of the development, and more abstract later on (Mundy and Sigman, 2006). Thus, JA is seen as a precursor to the development of theory of mind—the ability to attribute mental states to self and others (Baron-Cohen, 1991; Tomasello, 1995; Charman et al., 2000). Indeed, current research shows a relation between JA and theory of mind (Moll and Tomasello, 2004), language development (Brooks and Meltzoff, 2005), and overall social competence at later developmental stages (Mundy and Sigman, 2006).

It is argued that as the child develops, the ability to coordinate attention becomes internalized and the capacity to socially coordinate mental attention with internal representations appears, thereby playing a central role in the development of social cognition (Mundy and Jarrold, 2010). As children develop, they acquire the ability to coordinate attention not only to overt aspects of the environment such as objects or people, but also to covert aspects such as ideas, intentions, and emotions (Tomasello, 1995; Adamson et al., 2004; Mundy and Sigman, 2006; Van-Hecke et al., 2007). We would like to suggest that this developmental process may also reflect internalizations of cultural values and community expectations.

It is long known that the individual development is a product of a complex interaction between personal characteristics and environmental contexts. Through the first several years of childhood, the child's cognizance of his culture evolves and manifests itself through his behavior and internal processes (Dunn, 1994). Thus, when assessing JA it seems essential to take into account the context of the interaction and the child's ecology (Cole and Bruner, 1971).

Societal foundations, which reflect, among other things, values, community type, and socio-economic resources, correlate with a variety of developmental, cognitive and socio-emotional outcomes (Zill et al., 1995; Lareau, 2003; Vigil et al., 2006; Bartkowski et al., 2008). Hence, it is plausible that the corner stone of social interaction, the parent–child interaction characteristics, would reflect these factors in some way. Rogoff's work pointed to the notion that adults and children from all cultures jointly structure their interaction and engage in social referencing, and underscored that social referencing reflects socio-cultural characteristics and cultural values (Rogoff, 1990). Specifically, an ethnographic research by Rogoff et al. (1993) pointed to the relation of socio-cultural variances to differences in JA characteristics, specifically noting culturally dependent differences in directing attention to non-verbal cues, such as shared gaze, changes in posture and affect to highlight a target object or event in the child's natural habitat (Rogoff et al., 2003).

Literature regarding intercultural differences in parent–child interactions emphasizes the role of transmission of cultural characteristics to children from the adult environment. It has been suggested that socio-cultural effects of JA may serve a particularly important role in the process of socio-emotional development since the ability to jointly attend and share experience in response to a culturally specific stimulus is thought of as a key arena for acquiring and integrating culture (Callaghan et al., 2011). We would like to suggest that JA may serve as an instrumental mechanism in these processes.

It has been recently suggested that as we develop, our sensitivity to socio-cultural variation and to contextual cues becomes apparent through the maturation of the ventral prefrontal cortex (VPFC) (Luna et al., 2001; Davidson et al., 2006). The VPFC is a brain region mediating the executive functions of valuation, inhibition and rule acquisition. As maturation processes occur, the child's sensitivity to context is enhanced, a process expressed by increased ability to exert inhibitory control, regulate orienting to targets in the environment (Harel et al., 2010; Geva et al., 2011), and express social rules adaptively (Nelson and Guyer, 2011). According to Nelson and Guyer (2011), these processes emerge at preschool age, advance markedly in middle childhood and continue to develop through adulthood. Thus, it seems that the enhanced ability to use socio-cultural rules in order to adaptively function in society is made possible by the interaction between internal long-term neuronal maturational processes and the exposure to socio-cultural stimuli in daily life. Moreover, maturational processes in the VPFC networks that emerge at preschool enable the child to express behaviorally their sensitivity to socio-cultural cues and ecological contexts.

Bronfenbrenner's classical ecological systems model (ESM, Bronfenbrenner, 1979) posits that children's development is affected by the various systems which surround them. This model's basic premise refers to five levels of context, concentrically organized around the child (1) the microsystem—the child's immediate environment; (2) the mesosystem—connections between microsystems or contexts; (3) the exosystem—wider factors with which the child has no direct contact, but nonetheless still impacts her/his life; (4) the macrosystem—the broad society to which the child belongs; (5) the chrono-system—changes and events over the life-course and socio-historical circumstances. Current research supports the notion proposed by the ESM (Odom et al., 2004; Jordan, 2005; Stacks, 2005). These lines of work show that factors from different ecological levels join together in scaffolding various personal characteristics and mechanisms, such as at the macrosystem level of ethnicity (Lewis et al., 2010), the exosystem level of religiosity (Bartkowski et al., 2008) and social-class (Hart and Risley, 1995; Lareau, 2003; Farkas, 2004), and the mesosystem and microsystem levels of familial construct (Perner et al., 1994).

Adherence to different cultural values may account for interpersonal differences in a variety of children's individual measures at various levels of the ESM. For example, in Asian societies, children who are shy and reticent are high on well-being measures as opposed to their Western counterparts, since these behaviors match the collective values of the Asian culture (Chen, 2000). At the intermediate ecological level, it was shown that adherence to religious values moderates the relation between familial conflict and children's pathological outcomes (Davis and Epkins, 2009).

Parent–child interactions and specifically JA are usually explored through the prisms of either the microsystem or the macrosystem. Our current base of knowledge regarding JA is mainly based on microsystem variables that pertain to the biological or temperamental factors influencing JA (Mundy and Neal, 2000; Van-Hecke et al., 2007; Mundy and Jarrold, 2010). For example, these studies emphasize the child's degree of communicational competency or maternal characteristics as predictors of JA (Kasari et al., 1990; Gaffan et al., 2010). It was also found that overall gender does not predict JA in and of itself (Mundy et al., 2007). Research also points to macrosystem level effects, by showing intercultural differences in JA mediated processes. For example, differences were found between Western and Non-Western societies in JA behaviors. Thus it was shown, that in western societies JA seems to be directed more by the child, as opposed to non-western societies in which JA is more parent-directed (Vigil, 2002). This phenomenon was explained by the different belief systems between the Chinese culture and the British culture regarding the child's abilities and needs (Vigil, 2002).

The reference to JA in regard to the mesosystem and exosystem is scarce in current literature, although cultural values are expected to be expressed at these levels as well. It is known that developmental variations exist in ESM's intermediate levels, such as the effect of socio-economic status (SES) in a certain ethnic community. These variations are evident in the academic area (Zill et al., 1995) and the socio-emotional area (Bradley and Corwyn, 2002; Zhang et al., 2009). Similarly, different developmental norms were found among rural and urban communities in measures such as motor development, speech, visuo-spatial processes (Polyakov, 2008), and adaptive living skills (Bornstein et al., 2005). The current study intends to add to this knowledge base concerning intermediate-scale societal variations and highlight the potential relations between different ecological contexts and dyadic JA.

An important component to be considered in this context is that of gender. The relation between cultural characteristics and individual measures might be moderated by the child's gender. All in all, boys and girls seem to show differences in interactional styles from very early on. For example, empirical research has shown that from toddlerhood, girls tend to express more affect than boys (Dunn et al., 1987). It is also evident that boys are encouraged toward self-assertion while girls are encouraged to show social orientation (Lanvers, 2004). At preschool age, children also generally show enhanced gender stereotyping, and preference for specific toys, i.e., household games for girls and construction games for boys (Desouza and Czerniak, 2002). The degree of attunement to gender-roles is an integral part of any given culture, thus, we may postulate that the aforementioned sensitivity to cultural norms and behaviors is also relevant to the realm of traditional sex-roles.

Societies differ in the degree of adherence to traditional gender roles. As such parent–child interaction differences may reflect culture-dependent promotion of gender roles to different extents. This has been shown at the marcosystem level. For example, non-western cultures have been noted to promote traditional sex-roles to a larger extent than western cultures, evident both in adults and in children (Williams and Best, 1990). This variation is explained by the social-cognitive theory (Bussey and Bandura, 1999) that underlines modeling, enactive experience, and direct tuition as underlying mechanisms in gender role-acquirement. As such, it may be also sensitive to finer socio-cultural cues, such as to exosystem level variables.

The current paper is guided by the framework that macro and micro socio-cultural characteristics play an integral role in parental acclimation of infants to their respective culture by affecting central characteristics of parent–child interaction. This shaping process may be expressed as subtle second-to-second changes in specific gaze cues, posture and affect-regulation dynamics during spontaneous social child-caretaker interactions and be evident even as a function of fine socio-cultural differences.

The Israeli society is highly heterogeneous and is characterized by marked differences at the macro-level. Most socio-cultural research of the Israeli society is concentrated, as in other societies, in understanding the effects of macro-level variables (Birnbaum et al., 2010), while the effects of finer socio-cultural processes on parent–child interaction are yet under-studied. This project was focused on studying differences at the exosystem level, while examining the specificity of the relations between sociocultural characteristics and JA. We sought to study the degree of specificity of this relationship by expecting differences in JA, even when macrosystem level variables are held constant. As such, exosystem level differences are noted within the main-stream community, which is characterized by middle-SES, Jewish religion, with a stable conservative family structure. Three different communities characterize this society (Lavee and Katz, 2003): (1) Secular families that identify themselves less with religious values. Children from these families are educated in institutions that encourage western individualism and a sense of self-fulfillment and exploration; (2) Families belonging to religious communities that also endorse liberal values. Children from these families typically attend religious institutions that emphasize traditional values, but typically also cultivate western modes of thought. These communities highly value the role of shared familial learning, which is an integral part of their daily life; (3) Families belonging to religious communities and are affiliated with institutions that mainly promote traditional and conservative values especially concerning gender. Children from these families grow up in families that hold and encourage traditional familial roles.

We would like to propose, that similar to the finding that show that JA is the mechanism mediating between values and behavior at the macrosystem (Rogoff et al., 1993, 2003), these processes occur also at the exosystem and the mesosystem. Namely, the variations in the value-systems in our sample would manifest themselves in dyadic JA processes as well. The aim of the current study was to assess the effects of adherence to cultural values on the manifestation of JA at 5 years of age (kindergarten age), with a special emphasis on the type of stimuli the child was presented with (varying in social load). We assumed that JA initiation would manifest itself as a function of the toy's social load.

Our primary hypotheses were, first, that overall, irrespective of cultural differences, JA would increase in context of interaction with a social toy relative to a non-social task. This hypothesis is in line with accumulated research that shows an unequivocal preference of humans to orient toward faces and other social stimuli relatively to non-social ones (Bard et al., 1992; Valenza et al., 1996).

Secondly, we hypothesized that community characteristics would be correlated with the initiation of JA among participants. Specifically, we assumed, given that traditional societies cherish family and community values, these societies would tend to share attention in response to stereotypical value-carrying stimuli. Thus, we presumed that more bids for JA would be made among children from traditional communities toward the social stimuli presented. Furthermore, we hypothesized that JA initiation would be explained by gender differences, whereby girls would tend to share the social aspect of stimuli more than boys with their social partner. This hypothesis is based on the notion that girls are known to be generally more socially oriented than boys (Romer et al., 2011).

Specific hypotheses concerning the interactional effects were as follows: (1) an interaction between gender and type of stimulus, so that girls would be more inclined to initiate JA to the social aspects of the stimuli presented (Romer et al., 2011); (2) a triple order interaction between gender, adherence to tradition and type of stimulus, so that girls from more adherent communities would be more inclined to initiate JA to the social aspects of the stimuli presented (Williams and Best, 1990).

As for the parental educational factor of the exosystem level, differences within each of the three communities may be also noted in the level of parental formal education. Parental education has been found to predict various socio-emotional outcomes, including social-referencing and specifically JA (Mundy et al., 2007) and are thus hypothesized to moderate the socio-cultural effect. We hypothesized a moderating role in the relationship between community factors and the initiation of JA sharing to social stimuli.

Finally, to explore the degree of specificity of the effects of culture on behavior, we hypothesized that the differences in JA will be corroborated, to some degree, by differences in affect. In line with work showing a tendency for the initiation of JA to be accompanied by positive affect (Bakeman and Adamson, 1984; Mundy and Acra, 2006), we also postulated similar effects with mean time of positive affect as a dependant variable, so that children will tend to show more positive affect when presented with social stimuli, moderated by gender, community, and education factors.

## Materials and methods

### Participants

This research is part of a large-scale longitudinal study on child development focusing on 5–7 year olds. The current research included 62 children (33 boys, 29 girls) who were assessed at 5 years of age (*M* = 5.5 years ± 1 month). All families were Jewish, Caucasian, rated as middle SES, with a conservative family structure. All children in the sample attended kindergartens in their respective communities. The cohort consisted of families varying in level of adherence to tradition, defined by religious status (2 levels) and community type (3 levels), and level of parental education (2 levels). The sample was classified based on religiousness and education, as suggested by Friedman (2011). In order to define the degree of familial tradition and education level, the following indices were created: (1) Adherence to tradition index (ATI)—an index score composed of the families' degree of religiousness and the social composition of the familial community, eliciting three levels of tradition—low, medium, and high. The Low-ATI level group was comprised of families who define themselves as secular—not having a religious ties or traditional practices, and living in urban areas with high access to heterogeneous central population-concentrations, characterized by western values. The Intermediate-ATI level group comprised of families who lead a religious lifestyle, but their community of origin also promotes high tolerant, western standards. These families live in a small rural town in Israel (~2500 residents) revolving around similar ideological and cultural elements, thus creating a homogenous sub-sample (IBS, 2010). The High ATI Level group is comprised of families who lead a religious lifestyle and are connected to religious communities who advocate high adherence to traditional life style and shy away from ideological western values; (2) Parental education index—this score was comprised based on the lower level of education attained by the mother or the father. Scores were classified into two levels—low education by Israeli standards, (i.e., no academic degree) and high education (i.e., at least a bachelor's degree or higher).

### Procedure

Children and their parents were invited for testing in the laboratory at 5 years of age. Upon arrival, parents provided informed consent, followed by a warm-up period in the testing room, during which the parents filled out a number of questionnaires assessing demographic information and the child became more comfortable in the testing environment and with the experimenter.

Following this period, the parent and child engaged in a focused attention task designed to assess attention behaviors. The child was seated in front of a table while an experimenter, positioned at a 90° angle from the child, presented one of four toys varying in social load for free exploration: a high social load toy was a doll with a doctor's kit; a Mr. Potato Head^©^ with various accessories, and Lego^©^ blocks with miniature figurines, representing intermediate social load; and a construction game with no social elements, Kinex^©^, acting as a low social load toy. Meanwhile, the parent was seated farther away from the table and was instructed to allow the child free exploration and action with the toys. This 10 min long procedure was divided into four blocks, each lasting 2 min, intermitted by 30 s long breaks. The task was modified from a procedure by Ruff and Capozzoli (2003), by including a set of distracters presented during the dyadic interaction, rendering it into a more complex one. This modification to the paradigm was carried out in order to provide a more dynamic environment for the child, so as to encourage engagement in JA (Kirlik et al., 1993; Leavens and Bard, 2011). In three of the trials, 4 s long visual, auditory, and audio-visual distracters were presented in the background while the child played with the target toy. The order of the toy and distracter presentation was randomly set and counterbalanced between participants. The entire procedure was videotaped for later coding. In the final part of the session mental age was assessed using Griffiths Mental Development Scales (GMDS-ER; Griffiths, 1993) in order to ensure typical development.

The main dependent measures analyzed were the number of times the child directed or redirected the adult's attention to an aspect of the toy, which were coded as joint attentional bids—(JABS) (Carpenter et al., 1998), and the time intervals in which the child showed affective responses during the trials. Inter-rater reliability was calculated based on 5% of the total sample, in which these participant's tapes were coded separately by a second trained rater. Cohen's Kappa was calculated for the JABS and positive affect measures, showing a 0.84 agreement rate for the JABS measure, and a 0.77 agreement rate for affect, supporting a sufficiently high reliability of the measures.

## Results

Mean comparisons of demographical characteristics for children and their parents, showed no significant differences between the three ATI levels on any of the measures of age at test, mental age; on neonatal measures of gestation age and birth weight, and on familial measures of maternal and parental education (Table 1).

**Table 1:** **Demographic characteristics (mean ± standard errors) of the participants in each group**.

	**High ATI (*N* = 17)**	**Mid ATI (*N* = 21)**	**Low ATI (*N* = 24)**	***p***
Gender (% F)	35	52	50	NS
Age at test (m)	66.38±0.92	64.07±0.73	65.81±0.69	NS
Mental age (m)	69.18±1.7	68.48±1.46	70.38±1.25	NS
GA (w)	36.48±0.9	37.22±0.88	37.05±0.78	NS
Birth weight (g)	2566.07±251.46	2656.28±211.18	2671.83±185.49	NS
Mat. educ. level	4.18±0.2	4.09±0.21	4.25±0.16	NS
Pat. educ. level	3.71±0.22	4.19±0.23	4.14± 0.17	NS

GA, gestational age; Mat. educ., maternal education; Pat. educ., paternal education.

### Joint attentional bids (JABS)

In order to test the effects of the independent factors on children's JABS and expressed affect, measures of JA were analyzed with a 3 × 3 × 2 × 2 mixed design analysis of variance (ANOVA), using toy social load (high, medium, low), degree of community ATI (high, medium, low), gender, and parental education index (high\low) as independent variables. The effects for JABS are presented in Table 2.

**Table 2:** **Tests of within- and between-subjects' effects on number of JABS**.

**Source**	***F***	***P***	**Partial eta squared**	**Observed power**
TSL	24.94	**<0.001**	0.504	1.00
TSL × Gender	4.06	**0.023**	0.142	0.696
TSL × ATI level	1.19	0.322	0.045	0.360
TSL × Education level	1.11	0.338	0.043	0.234
TSL × Gender × ATI level	3.61	**0.009**	0.126	0.860
TSL × Gender × Education	5.36	**0.008**	0.179	0.818
TSL × ATI level × Education	3.55	**0.010**	0.124	0.853

The bold values show the precise significance level. Computed using alpha = 0.05; TSL, toy social load; ATI, adherence to tradition index.

Results, presented in Table 2, show a main effect for type of stimulus. In order to identify the source of this effect, a within-subject *post-hoc* test was carried out. This analysis showed the frequency of JABS was three times higher when the child engaged with social stimuli (*M* = 1.86 ± 0.22 for the high social load, *M* = 1.91 ± 0.26 for the medium social load relatively to non-social stimuli (*M* = 0.63 ± 0.12), thereby supporting the first hypothesis that JABS increase as a function of social content. This analysis, detailed in Table 2 also yielded support for the effects of the following interactions.

### Type of toy × gender

**Figure 1:**
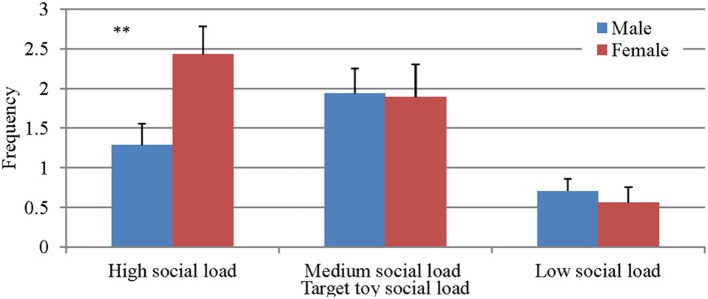
**JABS frequencies as a function of gender and social load**. ^**^p < 0.05.

An interaction of Type of toy × Gender was found, *F*_(2, 49)_ = 4.06, *p* < 0.05, η^2^ = 0.142. *Post-hoc* analysis revealed that the interaction is due to significant differences between girls and boys when presented with the highest level of social load so that girls initiated more JA than boys in the high social load toy context (Figure 1).

### Type of toy × community × gender

**Figure 2:**
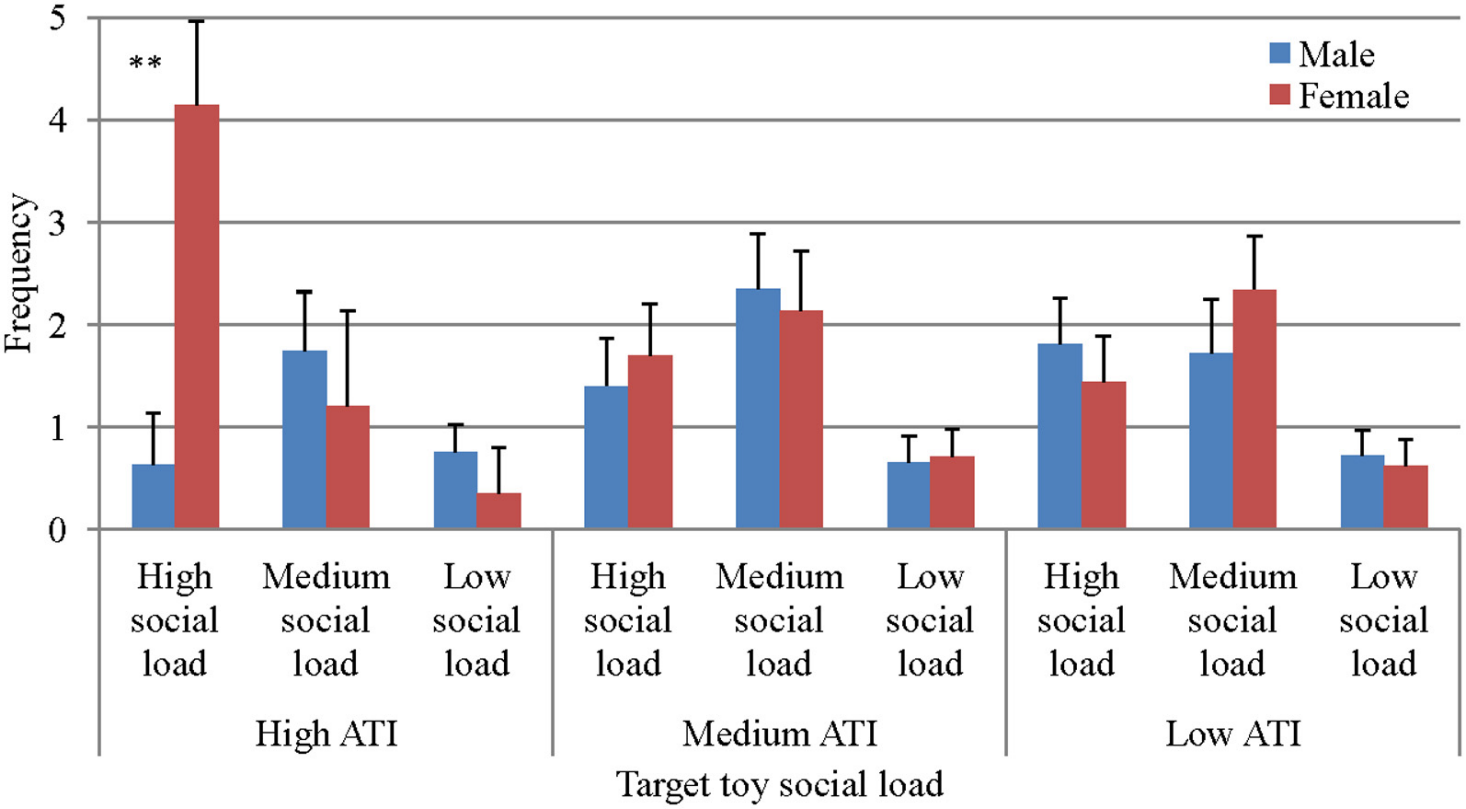
**JABS frequency as a function of gender, ATI, and social load (SL)**. ^**^p < 0.05.

A triple interaction of Type of toy social load × Community × Gender was also found, *F*_(4, 100)_ = 3.61, *p* < 0.01, η^2^ = 0.126 (Figure 2). *Post-hoc* simple effects analysis shows that in the highest level of ATI, a significant difference between boys and girls was evident, with the presentation of the highest level of social load. Girls from the High-ATI level made eight times more attempts to create JA than boys when presented with the most social stimulus, thereby supporting the second hypothesis.

### Type of toy × gender × education

**Figure 3:**
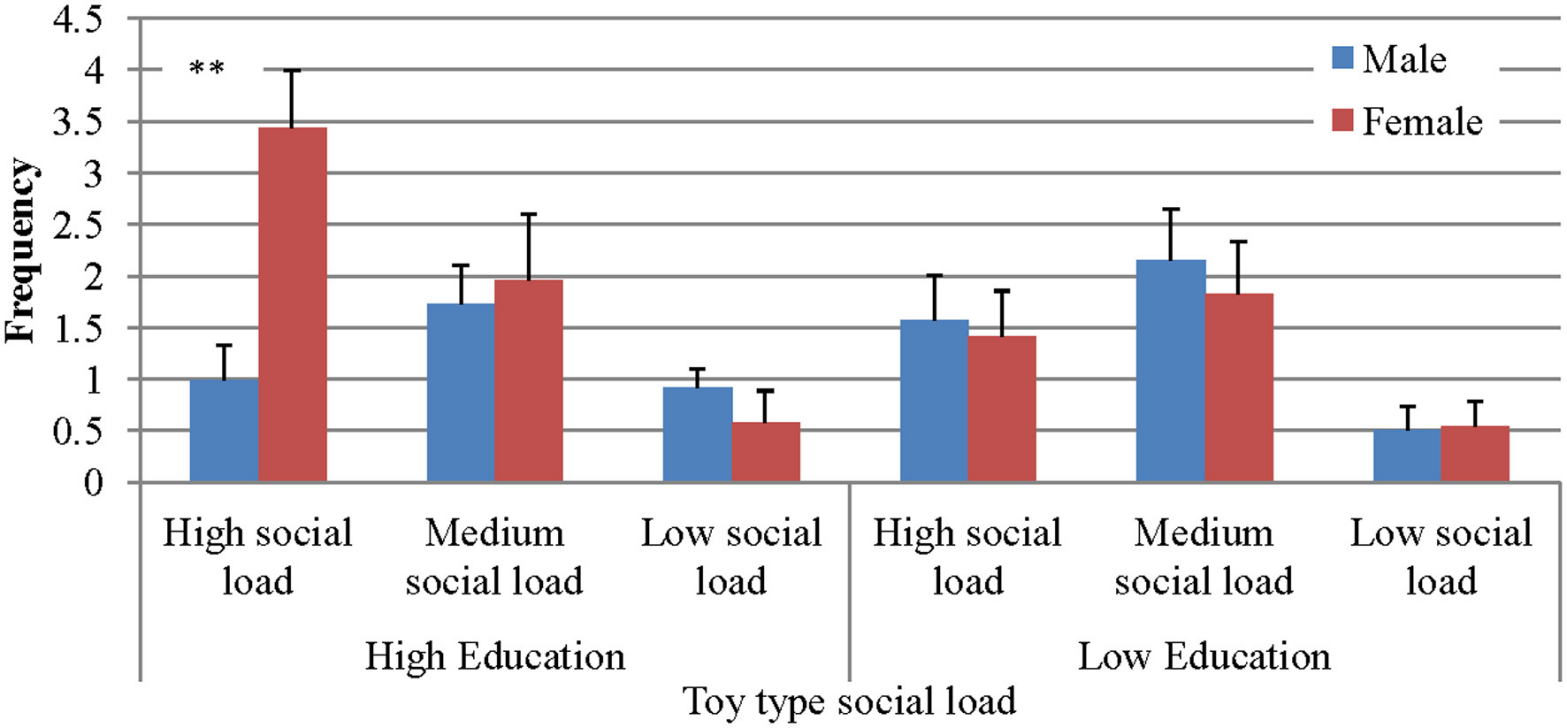
**JABS frequencies as a function of gender, education level, and social load**. ^**^p < 0.05.

A second triple interaction, Type of toy × Gender × Education, *F*_(2, 49)_ = 5.36, *p* < 0.01, η^2^ = 0.179 was also found (Figure 3). The interaction was such that girls in families with higher formal education initiated three times more JABS than boys in families with high education, when presented with the high social load toys. It seems, then, that the education factor augments the interaction effects of Gender × Type of toy, specifically in the high social-load condition.

### Type of toy × community × education

The third triple interaction of Type of toy × Community × Education interaction was also supported, *F*_(4, 100)_ = 3.55, *p* = 0.01, η^2^ = 0.124. However, this finding should be treated with caution and was not followed by post hoc tests in view of unequal cell sizes, particularly due to lack of low education intermediate ATI families.

**Figure 4:**
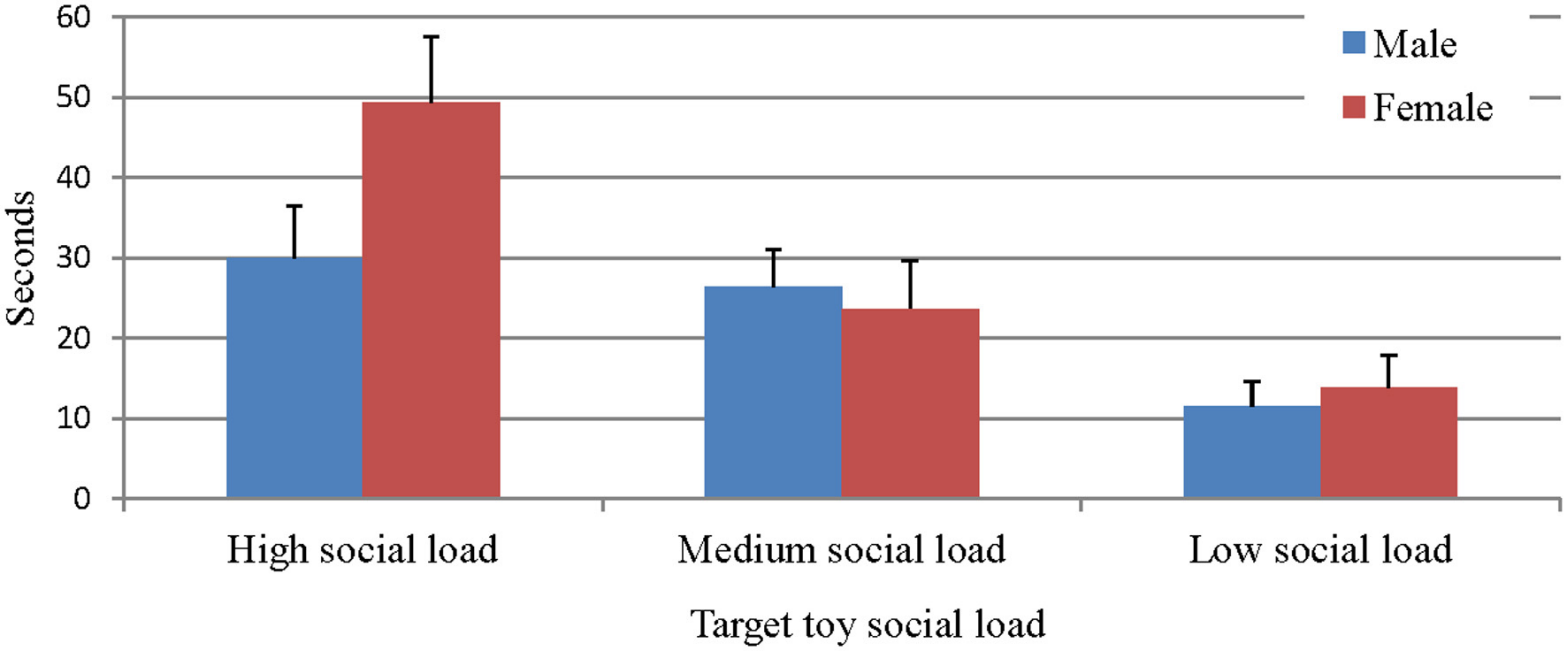
**Time of positive affect as a function of gender and social load**.

In order to test if similar effects would be found with affect as a dependant variable, the measure of affect was analyzed using a mixed-design ANOVA with the same independent variables. Results showed that positive affect increased as a function of social load, *F*_(2, 49)_ = 22.55, *p* < 0.001, η^2^ = 0.479 (Figure 4).

A within-subjects *post-hoc* test revealed that children spent the longest periods of time expressing positive emotions in the high social load toy condition (*M* = 39.67 ± 5.27), less so when presented with the intermediate level of social load context (*M* = 25.08 ± 3.76), and the shortest periods occurred when presented with the lowest level of social load (*M* = 12.70 ± 2.52). The main effect found with positive affect as a dependent variable supports the hypothesis concerning affect, which assumed children would be inclined to show more positive expression when presented with the social stimuli.

Moreover, a significant Type of toy × Gender interaction effect on positive affect was found, *F*_(2, 49)_ = 4.87, *p* < 0.05, η^2^ = 0.166, also shown in Figure 4. This analysis suggests that the aforementioned differences are more pronounced in girls than in boys. No significant effects regarding positive affect were found for ATI or education level.

In summary, JABS findings support the main hypothesis concerning JA initiation, showing that overall children indeed tend to initiate more shared attention and express more positive affect when presented with the social stimuli. Support was also found for the hypotheses concerning gender and community interactions with degree of social load, so that the tendency to initiate JA to different stimuli was differential among boys and girls from different ATI levels. Girls, particularly from High ATI communities and high parental education levels, were most affected by the interaction between community and toy load, in which circumstances they initiated most JABS and positive affect. Thus, it may be concluded that ATI level and parental education moderate the relation between gender and social load, explaining a significantly larger portion of the variation.

## Discussion

The objective of this study was to broaden the understanding of socio-cultural factors effects on individual behaviors. The social behavior of monitoring a partner's attention to a target was explored at the broad context of societal elements, holding the theoretical stance that the cultural environment in which the individual develops seeps into the micro-processes of social interaction and the child's socio-emotional development. Results seem to be in line with this conceptualization, showing that JA initiation is related to socio-cultural factors in the child's surrounding environment. The interaction effects that were found in the current study point to a notion concerning to “how,” by showing that socio-cultural factors map on to affect second-to-second dynamics of children's JA.

The main effect found in our research first validated the notion that JA initiation increases in response to social stimuli. This finding fits well with the social-cognitive framework of JA that views social-learning as a prime goal in the development of JA behavior (Mundy and Acra, 2006). Qualitatively speaking, when playing with the doll, children engaged more in symbolic play and invited the social agents to take part in it. At the non-social contexts, children mainly chose to explore the toy by themselves and initiated less JA, indicating that they preferred less to share the experience with their partners. This finding is compatible with the notion that at the developmental phase of 5 years of age, children express social-interaction intentions by JA, rather than employ it, as at younger ages, to gain concrete information about the target (Tomasello, 1995; Adamson et al., 2004; Mundy and Sigman, 2006; Van-Hecke et al., 2007).

The unique contribution of the current work lays in the moderating roles of socio-cultural factors of this primary interactive behavior. Findings first highlight the notion that socio-ecological factors come into play in the development of JA by interacting with gender and with the level of social load in the task. Overall, gender was not shown to have an effect on JA initiation in and of itself. Yet, girls have shown the preference to initiate JA to social stimuli (i.e., doll) to a greater extent than boys. This finding is supported by literature that shows gender-related differences in toy and game preferences at these ages. It is established that girls tend to prefer playing social games and pretend-play (Desouza and Czerniak, 2002). Several works also show that when engaging in this form of play, girls tend to take on feminine familial roles, including caring and handling (Jones and Glenn, 1991), while boys engage more in construction games (Desouza and Czerniak, 2002). The current study extends this notion to gender differences in JA to high social toys, such as dolls.

Moreover, findings highlight the interaction between gender and socio-cultural features. Findings showed that girls from the highly traditional background were much more affected by the changes in the context in which they created JA, and showed a significant preference to initiating JA to a social stimulus. In this specific community, a “gender stereotypic” discrepancy in shared-attention preferences was augmented. Girls in high ATI as compared with boys in high ATI have shown a higher inclination to initiate JA in the face of highly social stimulus. These differences were not found in the lower ATI levels. Similar effects were shown when comparing between JA initiation preferences of boys and girls from various levels of parental education. This may suggest that girls', and to a lesser extent, boys' preferences to share experiences in the face of social stimuli are reinforced in families with adherent life styles to certain cultural values—traditional and\or academic. This interaction may indicate that JA plays a role in two processes:

1. Traditional societies promote stereotypic gender-roles to a larger extent than non-traditional societies (Williams and Best, 1990). The mechanism for this process is not necessarily one that involves directive teaching, but rather implicit second-to-second gaze exchanges, to provide socio-cultural cues.
2. Girls seem to be more attuned to socio-cultural contexts and to social expectations than boys, as also enabled and manifested by the earlier maturation of the VFPC among girls (Raznahan et al., 2010).

Taken together, the mechanisms involved in the development of children's social interactions, seem to incorporate parental and community characteristics, through the use of JA, which serves as a particularly potent mechanism, scaffolding children's representations of their socio-cultural environment. This mechanism is particularly apparent in girls, in ecologies that are characterized by high adherence to rules, be it through high formal parental education and/or high adherence to traditional conventions. The finding with regard to parental education is intriguing, and may enrich our understanding of the underlying processes involved in intergenerational transmission of cultural values. It has been previously shown that parents vary in their ability to successfully transmit social values (Willson and Sherkat, 1994). Parents with more socioeconomic resources, but not necessarily higher income, were shown to be better able to transmit their cultural values (Myers, 1996). It is probable that parental education has both direct and indirect effects on JABS, by shaping parental attunement to the child's moment-to-moment responses, enabling parents with higher education to implement JA more effectively in order to signal culturally-valued events. The present study may have deepened the understanding regarding the importance of attunement to various cultural value-systems at different levels of the child ecology. It seems that through shared attention to primary aspects of their ecology, children acquire information necessary for them to become adequate members of their society (Leont'ev, 1981; Cole, 1985; Vygotsky, 1987). Children consistently take roles in ongoing interactions and events in their communities, thus appropriating key-elements from their cultures through joint-exploration (Rogoff, 1990). The current study broadens the scope of accumulated knowledge in the field which established that these processes occur at the microsystem level (Mundy and Neal, 2000; Van-Hecke et al., 2007; Mundy and Jarrold, 2010) and the macrosystem level (Vigil, 2002; Vigil et al., 2006). The present findings extend this line to intermediate levels as well, showing that JA might be the mechanism connecting between cultural values and their behavioral manifestations at various ecological levels.

Overall, this research highlights the role of socio-cultural background in the regulation of JA behaviors in social interactions. Importantly, it underscores the potential of a qualitative analysis of JA processes, taking into account the nature of the object to which JA is initiated and the types of contexts and stimuli to which the individual's society of origin directs the sharing of attention and experience. Yet, the modest sample size used in this research (*N* = 62), warrants caution in the interpretation of the results. Moreover, the paradigm was conducted in a structured lab setting, to enable control over multiple environmental factors, a replication in natural settings may highlight further components that need to be explored. Moreover, the study is based on a specifically selected Israeli sample, which was further classified by community ATI as a function of place of residence, religiosity, and the value-system that the family's society revolves around (i.e., goal-orientation or society\family orientation). Pending replication in other samples, future research may continue to explore a wider, ecologically valid model of socio-cultural effects on socio-emotional interactions to better understand the evolving means of culturally mediated sharing of experiences.

### Conflict of interest statement

The authors declare that the research was conducted in the absence of any commercial or financial relationships that could be construed as a potential conflict of interest.
